# Robotic management of superior mesenteric artery syndrome

**DOI:** 10.1093/jscr/rjae190

**Published:** 2024-04-04

**Authors:** Santiago A Muñoz-Palomeque, Ariadna Tobar-Tinoco, Máximo V Torres-Guaicha, Tábata L Tinoco-Ortiz

**Affiliations:** General Surgery Department, Hospital Metropolitano, Quito 170508, Ecuador; General and Laparoscopic Surgery, Universidad Internacional del Ecuador, Quito 170411, Ecuador; Universidad UTE, Avenida Mariscal Sucre s/n y Mariana de Jesús, Quito 170129, Ecuador; General Surgery Department, Hospital Metropolitano, Quito 170508, Ecuador; Faculty of Medicine, Universidad Central del Ecuador, Quito 170136, Ecuador; General Surgery Department, Hospital Metropolitano, Quito 170508, Ecuador

**Keywords:** digestive system surgical procedures, duodenal obstruction, superior mesenteric artery syndrome, robotic surgical procedures, mesenteric artery, superior

## Abstract

Wilkie’s syndrome is an unusual cause of upper intestinal obstruction due to mechanical compression of the superior mesenteric artery (SMA) to the duodenum, with nonspecific symptoms, whose diagnosis is confirmed by angiotomography. Initially, the treatment is conservative to regain weight and restore mesenteric adipose tissue, associated with postural changes of the patient. If this fails, surgical treatment is indicated, being laparoscopic duodenojejunostomy described as the gold standard. Robotics’ assistance is feasible and safe to carry out the procedure. We present the case of a 21-year-old male patient who comes with stabbing abdominal pain and persistent postprandial vomiting that has caused weight loss of 11 kilograms in the last 2 years without apparent cause, associated with gastroesophageal reflux. During the procedure, we evidenced open diaphragmatic pillars and duodenal compression due to SMA, and robotic-assisted laparoscopic hyatoplasty + Nissen fundoplication + duodenojejunostomy were performed without complications, with excellent post-surgical results.

## Introduction

Superior mesenteric artery syndrome (SMAS) is an unusual cause of chronic upper intestinal obstruction, with an incidence between 0.012 and 2.4%, secondary to extrinsic vascular mechanical compression of the third portion of the duodenum in the aortomesenteric angle [1–3].

There is an association between rapid weight loss and the development of SMAS, especially during puberty, due to the decrease in intra-abdominal adipose tissue that separates the SMA from the aorta, which causes greater acuity of the angulation between both structures and consequent duodenal compression [4, 5].

Symptoms are nonspecific and variable, with patients most frequently presenting with early satiety, abdominal distension, nausea, vomiting, and postprandial abdominal pain, followed by anorexia and weight loss [3]. Pain is relieved in the prone position, left lateral decubitus or genupectoral position, by relaxing the pressure of the SMA on the duodenum [5].

The diagnosis is usually delayed due to lack of knowledge of the disease. Useful diagnostic modalities are computed tomography (CT) as a standard tool, and ultrasonography [3, 6]. CT with mesenteric angiography helps determine the measurement of the aortomesenteric angle, being the main anatomical characteristic of this syndrome the narrowing of the angle between the SMA and the aorta to ≤25°, being the normal 38 to 65° [6, 7]. This narrowing leads to compression of the third portion of the duodenum as it crosses between the aorta and the SMA, and may even result in compression of the left renal vein. The aortomesenteric distance is reduced from the normal 10–28 mm to 2–8 mm, and the severity of the symptoms is related to the aorta – SMA distance [8]. Therefore, an aortomesenteric angle less than 22° and a distance less than 8 mm on contrast-enhanced CT angiography are consistent with SMAS [7].

Treatment is initially conservative and its main objective is to regain weight to restore mesenteric adipose tissue and thus increase the aortomesenteric angle, including gastroduodenal decompression, correction of hydroelectrolyte balance, enteral tube feeding or parenteral nutrition, additional to postural changes with postprandial prone position to cause the SMA to move anteriorly. However, there is a risk of failure between 20% and 30% [3, 7, 9].

If conservative therapy fails, surgical treatment is recommended, with several procedures described, including release of the ligament of Treitz with duodenal descent (Strong’s procedure), infrarenal transposition of the SMA; laparoscopic gastrojejunostomy and duodenojejunostomy, with overall success rates between 80% and 100% [3, 7, 10–13]. The latter is currently considered the most accepted procedure, and additionally, the robotics’ technique is feasible and safe to perform [10].

### Case report

**Figure 1:**
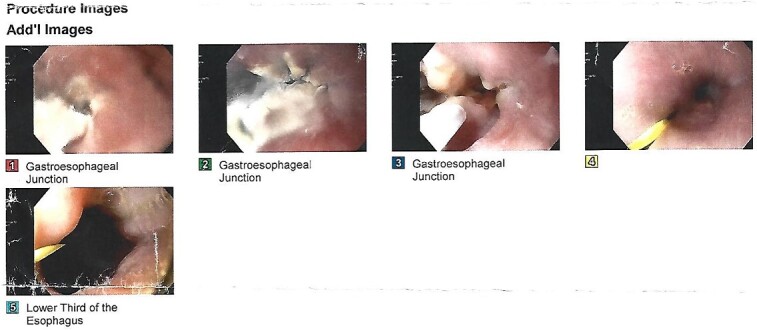
Upper digestive endoscopy.

**Figure 2:**
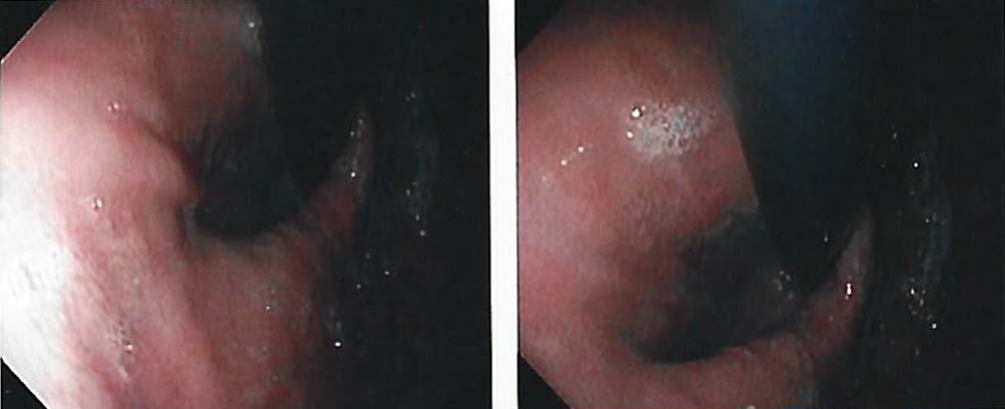
Upper digestive endoscopy with hiatal hernia.

**Figure 3:**
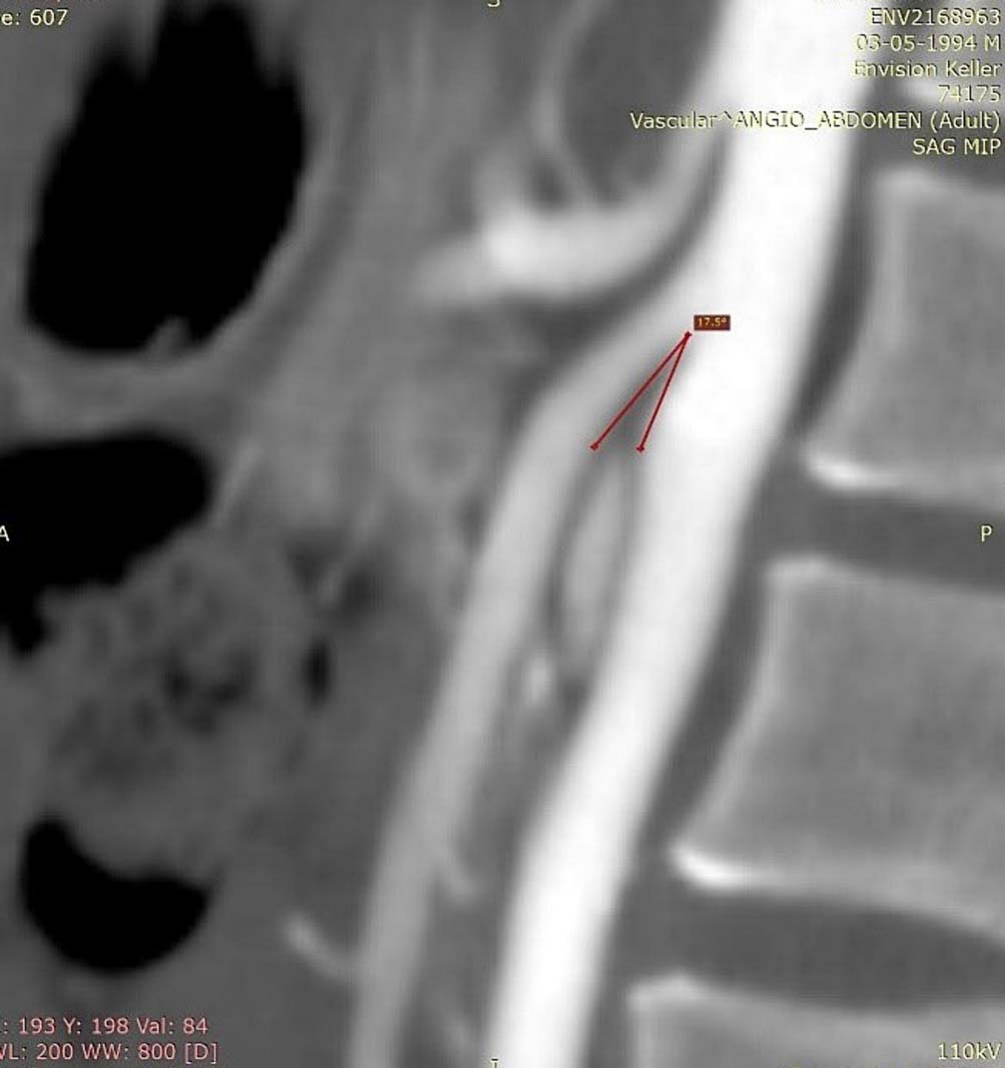
Computed tomography. Aortomesenteric angle of 17.5°.

**Figure 4:**
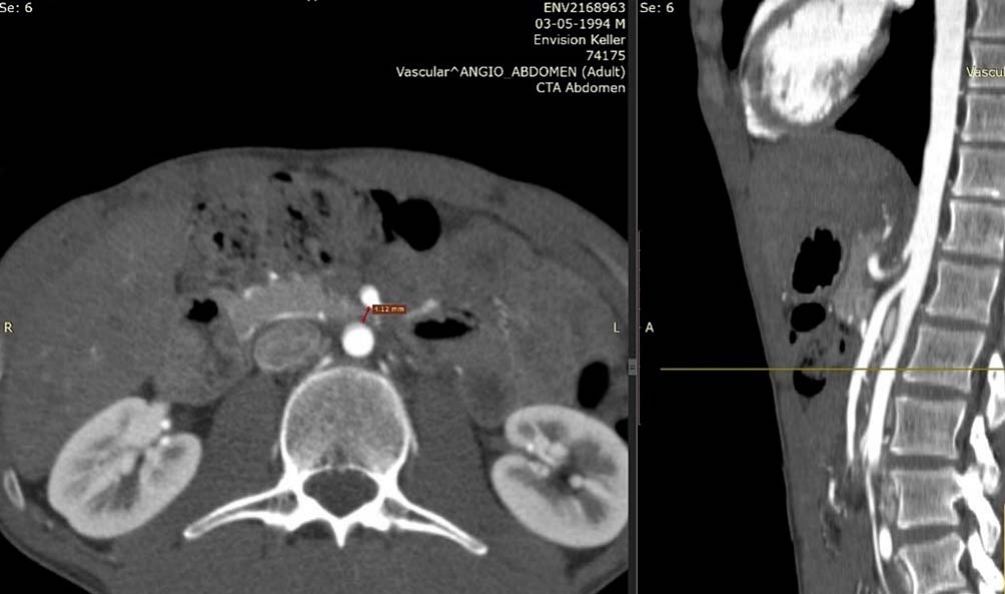
Computed tomography. Aortomesenteric distance of 4 mm. Wilkie’s syndrome.

We present the case of a 21-year-old male patient, with a history of laparoscopic cholecystectomy and 3 cryoablations for Barret’s esophagus with grade III dysplasia, who presented with persistent postprandial vomiting that had caused weight loss of 11 kg in the last 2 years without apparent cause, associated with gastroesophageal reflux that has worsened over time, occasional stabbing abdominal pain in the epigastrium, painful constipation, bloody stools and steatorrhea, managed clinically and symptomatologically without improvement in the condition. On evaluation, the patient was thin, with a BMI of 17.31 kg/m^2^, and in paraclinics, in endoscopy (Figs 1 and 2), Barret’s esophagus and hiatal hernia; In tomography (Figs 3 and 4), an aortomesenteric angle of 17.5°, with aortomesenteric distance of 4 mm.

**Figure 5:**
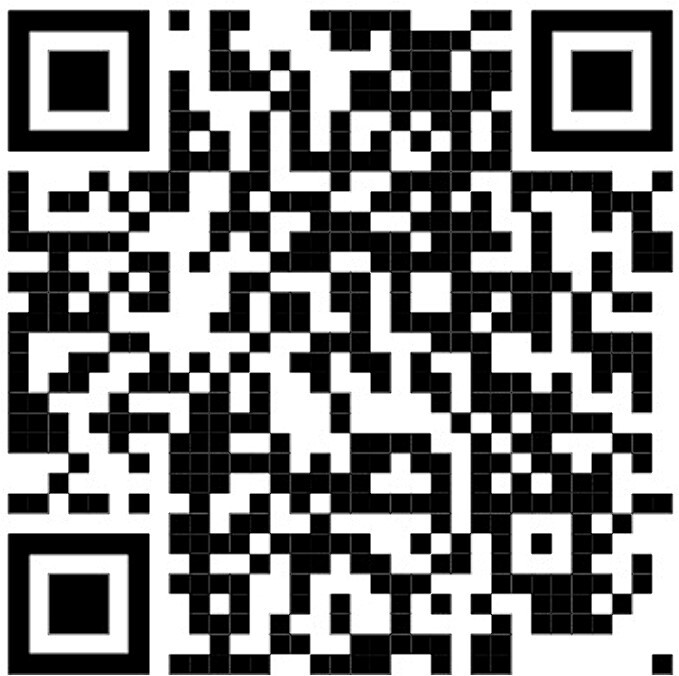
Surgical video.

Given the diagnosis of gastroesophageal reflux disease (GERD) with esophagitis + SAMS (Wilkie syndrome), surgical resolution was decided (Fig. 5), showing trans surgical findings of open diaphragmatic pillars, normal stomach, absence of duodenal distension, and presence of duodenal compression due to AMS. Finally, robotic-assisted laparoscopic anterior and posterior hyatoplasty + Nissen fundoplication + duodenojejunostomy were performed, without complications.

Subsequently, the patient had a favorable general evolution, absence of reflux symptoms, absence of pain, and adequate oral tolerance of the diet, for which medical discharge was indicated.

## Discussion

SAMS is a rare entity with a reported prevalence estimated between 0.013% and 0.3% [2, 7], with a median age of 23 years, and predominance in women over men with a ratio of 3 to 2 [3], which manifests itself with symptoms of chronic upper intestinal obstruction, evidenced in our patient, both in his age range and in the symptoms presented, in addition to gastroesophageal reflux.

Robotics’ surgical approaches for duodenojejunostomies have been described, with robotic surgery offering numerous advantages such as improved three-dimensional visualization, instrumented wrist articulation, and the surgeon’s ability to control multiple instruments and the camera, resulting in reduced operative times and faster recovery of the patient [7]. On the other hand, robotic duodenal surgery is a treatment option for many benign and low-grade malignant duodenal conditions not amenable to endoscopic intervention, which can avoid morbidity related to open surgery [14].

In this way, given that the symptoms altered the patient’s quality of life, and considering that in addition he had esophagitis due to GERD and hiatal hernia, the surgical procedure was decided by robotic laparoscopic route, which greatly facilitated the approach and the precision, without complications or incidents during them, reflected in the good post-surgical evolution and the early recovery of the patient.

During the evaluation of the patient with chronic upper intestinal obstruction, characterized by postprandial vomiting and recurrent abdominal pain, and who has also lost weight, SMAS is a diagnosis of exclusion that should be always considered as a probable etiology of intestinal obstruction, which will allow an early treatment of the pathology, avoiding its complications, as well as repercussions on the patient’s quality of life.

If conservative management fails, surgical management will be resorted to, recommending a laparoscopic duodenojejunostomy, since it has been demonstrated a high success rate with the lowest rate of associated post-operative complications, and robotics’ assistance can be considered in well-selected patients, with excellent short and long term results.
